# Supplementary material for: Time‑resolved multi-omic analysis of paclitaxel exposure in human iPSC‑derived sensory neurons unveils mechanisms of chemotherapy‑induced peripheral neuropathy
Source: Cell Death Dis. 2026 Feb 10;17(1):211. doi: 10.1038/s41419-026-08445-2 (PMC12921266; doi:10.1038/s41419-026-08445-2)
Supplement: Supplementary file 15 — Dataset 1. [file 41419_2026_8445_MOESM15_ESM.zip › CDD_Open_Data/04_RNA_Seq/Timeline_panel_plots_etc_2023-01-23_1609.zip!/Panel_Plots_etc/Evidence_Plots/48h/evidence_plot_standard contrasts_msigdb_hallmark_48h_PTX_vs_DMSO_ID5_M5924_HALLMARK_MTORC1_SIGNALING.pdf]

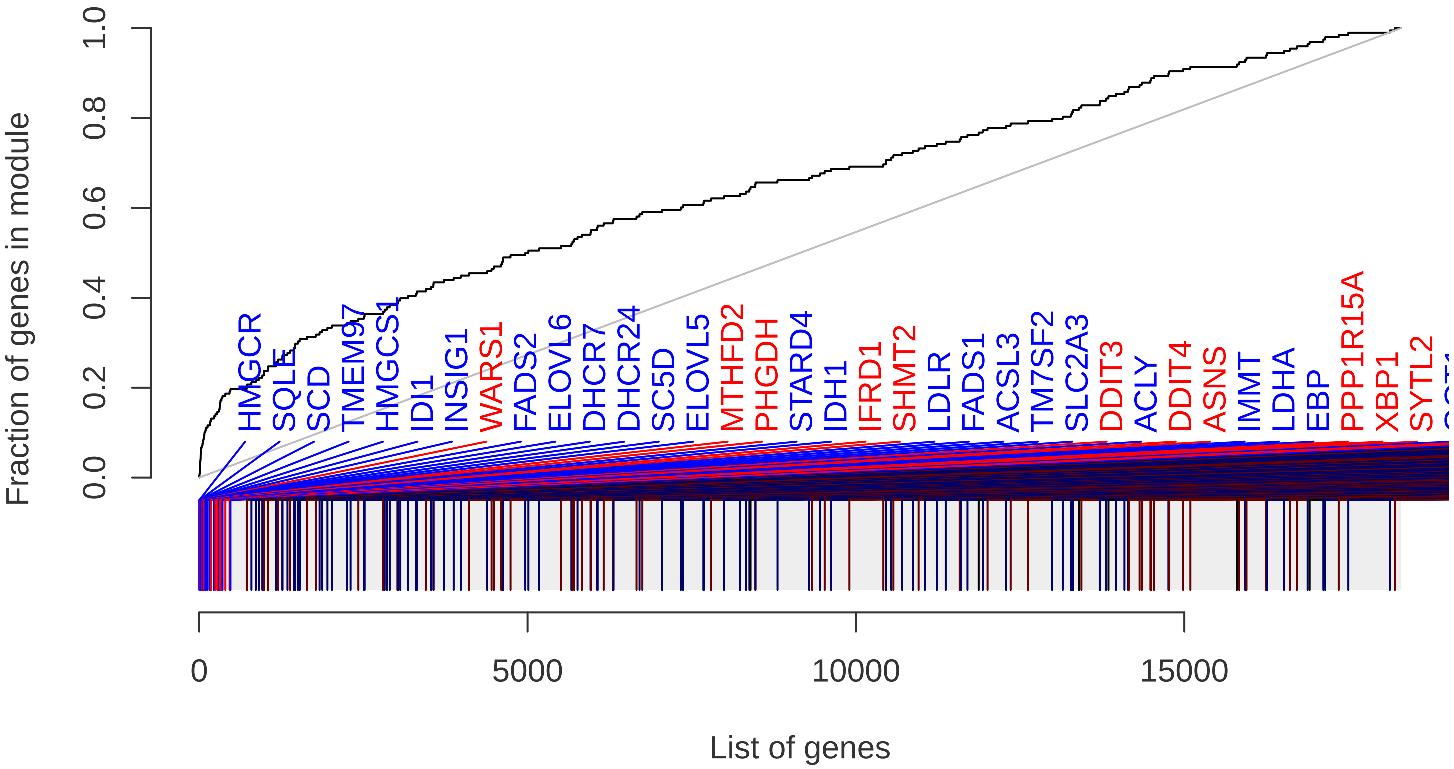

Fraction of genes in module
0.0
0.2
0.4
0.6
0.8
1.0
HMGCR
SQLE
SCD
TMEM97
HMGCS1
IDI1
INSIG1
WARS1
FADS2
ELOVL6
DHCR7
DHCR24
SC5D
ELOVL5
MTHFD2
PHGDH
STARD4
IDH1
IFRD1
SHMT2
LDLR
FADS1
ACSL3
TM7SF2
SLC2A3
DDIT3
ACLY
DDIT4
ASNS
IMMT
LDHA
EBP
PPP1R15A
XBP1
SYTL2
0
5000
10000
15000
List of genes
